# The phosphatase and tensin homologue deleted on chromosome 10 mediates radiosensitivity in head and neck cancer

**DOI:** 10.1038/sj.bjc.6605707

**Published:** 2010-05-25

**Authors:** W J Pattje, E Schuuring, M F Mastik, L Slagter-Menkema, M L Schrijvers, S Alessi, B F A M van der Laan, J L N Roodenburg, J A Langendijk, J E van der Wal

**Affiliations:** 1 Department of Radiation Oncology, University Medical Center Groningen, University of Groningen, Groningen, The Netherlands; 2 Department of Pathology and Medical Biology (HPC EA10), University Medical Center Groningen, University of Groningen, PO Box 30.001, Groningen 9700 RB, The Netherlands; 3 Department of Otorhinolaryngology and Head and Neck Surgery, University Medical Center Groningen, University of Groningen, Groningen, The Netherlands; 4 Sapienza–Università di Roma, Rome, Italy; 5 Department of Oral and Maxillofacial Surgery, University Medical Center Groningen, University of Groningen, Groningen, The Netherlands

**Keywords:** PTEN, HNSCC, radiosensitivity, locoregional control, EGFR pathway

## Abstract

**Background:**

For locally advanced squamous cell carcinoma of the head and neck (HNSCC), the recurrence rate after surgery and postoperative radiotherapy is between 20 and 40%, and the 5-year overall survival rate is ∼50%. Presently, no markers exist to accurately predict treatment outcome. Expression of proteins in the human epidermal growth factor receptor (EGFR) pathway has been reported as a prognostic marker in several types of cancer.

**Methods:**

The aim of this study was to investigate the prognostic value of proteins in the EGFR pathway in HNSCC. For this purpose, we collected surgically resected tissue of 140 locally advanced head and neck cancer patients, all treated with surgery and postoperative radiotherapy.

**Results:**

In a multivariate analysis, expression of the phosphatase and tensin homologue deleted on chromosome 10 (PTEN) was significantly related to worse locoregional control (LRC; HR: 2.2, 95% CI: 1.1–4.6; *P*=0.03), independent of lymph node metastases (HR: 5.6, 95% CI: 1.2–27.4; *P*=0.03) and extranodal spread (HR: 2.7; 95% CI: 1.2–6.5; *P*=0.02). *In vitro* clonogenic radiosensitivity assays confirmed that overexpression of PTEN resulted in increased radioresistance.

**Conclusion:**

Our study is the first report showing that expression of PTEN mediates radiosensitivity *in vitro* and that increased expression in advanced HNSCC predicts worse LRC.

The prognosis for patients with locally advanced head and neck squamous cell carcinoma (HNSCC) depends on numerous clinical and histopathological factors. Despite intensive curative treatment strategies, such as surgery and postoperative radiotherapy, 20–40% of all patients will develop a locoregional recurrence (LRR) and the 5-year overall survival rate remains ∼50% (Le Tourneau *et al*, 2008). For this reason, attempts have been made to identify molecular markers that are able to improve the identification of tumours that will develop LRR and thus might benefit from more aggressive treatment strategies (Partridge *et al*, 2005). One of the potential prognostic factors is the epidermal growth factor receptor (EGFR), which has been identified as an important prognostic marker in several other cancer types (Mellon *et al*, 1995; Fischer-Colbrie *et al*, 1997; Inada *et al*, 1999; Kersemaekers *et al*, 1999; Galizia *et al*, 2006). The human epidermal growth factor receptor (HER) family of tyrosine kinase receptors consists of four family members, of which the EGFR (EGFR/HER1/erbB1) and HER2 (HER2/NEU/erbB2) are the best known members (Rogers *et al*, 2005). Binding of the ligands for EGFR, such as TGF-*α* and EGF, to the extracellular domain of the receptor triggers a dimerisation of the receptors, resulting in autophosphorylation of the tyrosine-rich intracellular domain of EGFR. On activation, the receptors can either homodimerise or heterodimerise with other HER family members, such as HER2. This activated form of the receptor initiates two major downstream signalling pathways, the PI3K/AKT pathway and the Ras/Raf/MEK/ERK pathway. Activation of these pathways leads to increased proliferation, desensitisation to apoptosis and increased angiogenesis (Yarden and Sliwkowski, 2001; McKay and Morrison, 2007; Jiang and Liu, 2008).

A number of studies reported that overexpression of the EGFR protein (Grandis *et al*, 1998; Ang *et al*, 2002; Pivot *et al*, 2005) is associated with worse prognosis in HNSCC. However, in other studies, the association between the levels of EGFR expression and clinical outcome could not be confirmed (Eriksen *et al*, 2004; Smid *et al*, 2006; Fischer *et al*, 2008). There are various explanations for the differences between these studies, such as various antibodies, protocols and scoring methods. In addition, it has been shown that amplification of the EGFR gene is predictive for clinical outcome in HNSCC (Chung *et al*, 2006). As EGFR amplification analysis by fluorescent *in situ* hybridisation (FISH) is more reproducible than most immunohistochemical (IHC) staining procedures, this might prove to be a more valuable tool for prognosis than IHC.

The aim of this study was to investigate the prognostic value of EGFR, HER2 and their downstream pathway targets with regard to locoregional control (LRC) in locally advanced HNSCC. For this purpose, tumour specimens of HNSCC patients treated with surgery and postoperative radiotherapy were collected. In addition, we performed *in vitro* clonogenic survival assays to confirm the hypothesis that followed from the results of the clinical part of our study.

## Material and methods

### Patients and tissues

This study cohort was composed of consecutive patients diagnosed with HNSCC, uniformly treated with primary surgery and postoperative radiotherapy at the University Medical Center Groningen, The Netherlands, between 1993 and 2003. Clinical and histopathological data of all these patients were collected (*n*=198) with a follow-up of at least 3 years. Formalin-fixed paraffin-embedded surgically resected tissue of the primary tumour was collected and revised by an experienced pathologist (JEvdW). In 167 cases, sufficient tumour material was available to construct a tissue microarray (TMA). In this study, we included 140 patients for whom IHC stainings for all antigens were assessable. The pre-treatment characteristics of these patients are summarised in Table 1. In summary, the study cohort mainly consisted of male patients, with a median age of 60 years (range 24–90), with predominantly more advanced T-stage (T3–T4 in 74%), lymph node metastasis (64%) and advanced-stage (84% stage III and IV) cancers.

All patients underwent surgery of the primary tumour followed by postoperative radiotherapy. In 128 of the cases (91%), a neck dissection was performed. Postoperative radiotherapy was administered because of positive surgical margins (69%), lymph node metastases with extranodal spread (36%) and/or other adverse prognostic factors such as advanced T-stage, multiple lymph node metastases and/or perineural growth. Local recurrence was defined as a recurrence within 2 cm of the original tumour site and occurring within a 3-year period. Tumours arising more than 3 years after therapy were considered as second primary tumours.

### TMA construction

To construct TMAs, 5-*μ*m-thick sections were cut from paraffin-embedded formalin-fixed tissue blocks, slides were prepared and a standard haematoxylin and eosin (H and E) staining was performed. The representative regions in the tumour were marked on the H and E-stained slides by an experienced pathologist. Three cores of 0.6 mm diameter were taken from each donor block and put into the recipient paraffin block using the Manual Tissue Arrayer 1 (Beecher Instruments, Silver Spring, MD, USA). Six different normal tissue controls and one oropharyngeal SCC were included on each TMA block to ensure similarity of staining between the slides, orientation on the TMA and recognition of each TMA. Our series of 167 carcinomas was distributed on four TMAs. Sections (5 *μ*m thick) from each TMA block were cut and the first section was stained with H and E to confirm the presence of tumour cells in each core. Using TMAs with three cores with a diameter of 0.6 mm is considered to be representative for the heterogeneousness of the tumour (Camp *et al*, 2000; Kallioniemi *et al*, 2001).

### IHC staining

Tissue microarray sections were deparaffinised in xylene and rehydrated. Antigen retrieval was performed by heating in a microwave oven for 15 min in 10 mM citrate buffer (pH=6.0; for pERK, pAKT, PTEN); in EDTA (pH=8.0; for pEGFR); in a pressure cooker, three times for 5 min at 115°C (for PI3K); for 30 min at 95°C in a Tris buffer (pH=9.5; for EGFR); or for 30 min at 95°C in cell conditioner 1 buffer (Roche Diagnostics/Ventana, Basel, Switzerland) (for HER2).

After antigen retrieval, the endogenous peroxidase was blocked with a 0.3% peroxide solution. The following primary antibodies were used: EGFR (clone EGFR113, Novocastra (Newcastle upon Tyne, UK), ready to use), pEGFR (clone 1H12, Cell Signalling (Danvers, MA, USA), 1 : 200), PTEN (clone 6H2.1, Cascade (Winchester, MA, USA), 1 : 100), PI3K p110 (sc-1331, Santa Cruz Biotechnology, Santa Cruz, CA, USA, 1 : 50), pAKT (ser473) (clone 736E11, Cell Signalling, 1 : 50), pERK (clone 20G11, Cell Signalling, 1 : 50) and HER2 (clone CB11, Roche Diagnostics/Ventana, ready to use).

For immunodetection, we used appropriate biotinylated (PTEN, pEGFR, pERK) or horseradish peroxidase (HRP)-conjugated (PI3K) secondary antibody or Envision (Dako, Glostrup, Denmark) (pAKT) followed by appropriate HRP-conjugated tertiary antibody (PI3K) or HRP-conjugated streptavidin (PTEN, pEGFR, pERK) and developed with 3,3′-di-aminobenzidine chromogen solution (Dako) followed by a routine haematoxylin counterstaining.

### Interpretation of IHC

Staining intensity was semi-quantitatively scored as negative (0), weak positive (+), positive (++) and strong positive (+++) staining. For statistical analysis, any positive staining above background was considered as positive. In addition, the percentage of positive cells was recorded. In case of differences between cores, scores were averaged for statistical analyses.

For the EGFR and HER2 staining, no and incomplete membranous staining were considered negative, and only complete IHC staining of tumour cell membranes above the background was considered positive, independent of the intensity and percentage of positive cells according to the standard Herceptest protocol.

The cases were considered positive for the pEGFR staining if 20% or more of the cells had membranous, cytoplasmic or nuclear staining; positive for pAKT if 27.5% or more of the tumour cells had cytoplasmic staining; positive for PTEN when 7.5% or more of the tumour cells showed a cytoplasmic staining; and positive for PI3K when more than 35% of the tumour cells had cytoplasmic staining. The cutoff percentages are based on receiver operating characteristic curve analyses for LRR (data not shown) (Zweig and Campbell, 1993). The cases were considered positive for pERK if there was nuclear staining in any of the tumour cells.

Evaluation of immunostaining was performed independently by two observers without information on the clinical data. In case of discrepancies between the observers, cases were reviewed with an experienced pathologist and scored on consensus opinion. Only patients with at least two representative cores were included in the analysis.

### Fluorescent *in situ* hybridisation

Fluorescent *in situ* hybridisation analysis was used to determine copy number changes of the EGFR gene. Tissue microarray sections were deparaffinised in xylene and rehydrated, pre-treated in a pressure cooker at 120°C in a Tris/EDTA (pH 9.0) buffer, incubated with RNAse A and with 0.1% pepsin and dehydrated in an ethanol gradient. The DNA was denatured for 12 min at 80°C with the dual-colour EGFR/CEP7 FISH probes (Vysis LSI EGFR SpectrumOrange/CEP 7 SpectrumGreen probe from Abbott, Hoofddorp, The Netherlands) and hybridised at 37°C overnight. The sections were incubated in a 0.3% NP-40 solution at 73°C and a 0.1% NP-40 solution at RT. The sections were dehydrated and covered by a drop of DAPI (1 : 3000) in Vectashield (Vector Laboratories, Burlingame, CA, USA) and covered by a coverslip. Images were captured using a Leica DMRA2 fluorescence microscope (Leica Microsystems, Wetzlar, Germany) equipped with a Leica DC 350F charge-coupled device camera. Digital images were processed with Leica CW4000 software. Interphase nuclei were examined by eye and the orange and green signals were counted separately in ∼20 nuclei per core. The ratio between EGFR (orange) and centromere 7 signals provides an accurate estimation of copy number differences. A ratio of 1.0 indicates normal copy (or polyploidy of chromosome 7 if >3 signals of both probes are observed per nucleus), a ratio <1 is loss and >2 is gain. Nuclei were considered to contain amplification of EGFR when the number of signals exceeded five (and ratio >2.5).

### Statistical analysis

Statistical analysis was carried out using the SPSS 14.0.0 software package (SPSS Inc., Chicago, IL, USA). Association between different markers and between markers and clinicopathological characteristics were performed using the *χ*^2^-test. The primary end point used in this study was LRC, which was defined by the time from surgery until the first LRR in the case of an event or from the time from surgery until the last follow-up date in the case of no event.

For the univariate and multivariate analysis of clinical outcome, a Cox regression analysis was used. The categorised covariates that showed a trend (*P*<0.10) in the univariate analysis were put into a back-step multivariate Cox regression analysis. *P*-values <0.05 were considered significant. Kaplan–Meier survival curves for LRC were created to illustrate the differences.

### Cell culture and transfection

The Hek293 cell line was cultured in DMEM (1 g l^−1^ glucose) with 10% fetal bovine serum, 2 mM ultra-glutamine, penicillin and streptomycin (all purchased from BioWhittaker, Basel, Switzerland). The cells were transfected using Fugene 6 reagent (Roche Allied Sciences, Almere, The Netherlands), as described by the manufacturer, with the empty vector pcDNA3 or the pcDNA3–GFP–PTEN (Addgene plasmid 10759). From one day after transfection on, the cells were cultured in DMEM containing 800 *μ*g ml^−1^ Geneticin (Invitrogen, Carlsbad, CA, USA). Geneticin-resistant colonies were separately cultured, and the expression of GFP/PTEN was confirmed using FACS analysis for GFP and immunostaining on western blot using an antibody for PTEN (clone 28H6, 1 : 500; Santa Cruz Biotechnology).

### Clonogenic survival assay to determine radiation response *in vitro*

The 96-well plate clonogenic assay based on limiting dilutions was used as described previously in detail (Grenman *et al*, 1989). The data are based on the average of three independent experiments.

## Results

To assess the expression levels of proteins and phosphoprotein isoforms having an active role in the EGFR pathway in locally advanced HNSCC, IHC was performed on TMAs containing primary tumour material from patients with HNSCC who were all treated with primary surgery and postoperative radiotherapy. In 140 HNSCCs, EGFR was positive in 17 cases (12%), pEGFR in 56 cases (40%), PI3K in 53 cases (38%), PTEN in 43 cases (31%), pAKT in 35 cases (25%), pERK in 22 cases (16%) and HER2 in 11 cases (8%) (See examples in Supplementary Figure 1).

### PTEN expression is associated with EGFR activation

To determine whether the activation of the EGFR pathway resulted in the simultaneous activation of other downstream EGFR pathway targets, we performed cross-table analysis between all targets (Table 2). A significant association was found between the expression of EGFR and its activated isoform (pEGFR) (*P*=0.027) (Table 2a). In addition, pEGFR staining and pERK staining were found to be associated (*P*=0.001) (Table 2b). The pERK staining showed an inverse relationship with the PI3K staining (*P*=0.004) (Table 2c) and a positive correlation with PTEN-positive staining (*P*=0.028) (Table 2d). Positive staining for PI3K was associated with positive staining for HER2 (*P*=0.028) (Table 2e). Surprisingly, a positive pAKT staining showed a positive association with PTEN positivity (*P*<0.001) (Table 2f). Combinations of other stainings were not associated significantly (Supplementary Table 1).

### EGFR expression is associated with more advanced-stage tumours

The correlations between IHC staining and clinicopathological characteristics showed that tumours with a high percentage of pAKT-positive cells had an increased risk on lymph node metastases with extranodal spread (*P*=0.011). High expression of EGFR correlated with lower T-stages (T1–T2) (*χ*^2^=4.6; *P*=0.032) and with lower stages (stage I–III) (*χ*^2^=9.2; *P*=0.002). For the other clinicopathological characteristics, no associations were found with any of the IHC stainings (Supplementary Table 1).

### EGFR expression is strongly associated with DNA amplification

We found high-copy DNA amplification of the EGFR gene in 14 out of the 118 assessable cases (12%). Immunohistochemical EGFR protein expression was significantly associated with DNA amplification (*χ*^2^=85.22; *P*<0.0001) as 13 of 16 positive cases showed amplification in contrast to only 1 of 102 EGFR-negative cases. The IHC staining for EGFR was shown to be highly specific (97%) and highly sensitive (93%) for EGFR amplification (Table 3). Interestingly, the only case with EGFR amplification that was scored IHC negative (because of lack of membranous staining) showed the strongest cytoplasmic staining among the other cases.

### PTEN predicts LRC

**Figure 1:**
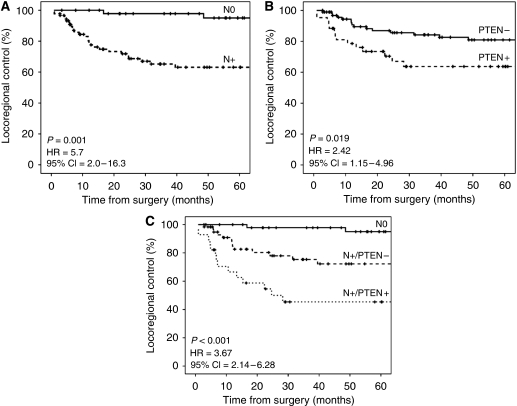
Kaplan–Meier analysis for locoregional control. (**A**) Comparing N0 with N+ cases. (**B**) Comparing phosphatase and tensin homologue deleted on chromosome 10 (PTEN)− with PTEN+ cases. (**C**) Comparing N0 with N+/PTEN− and N+/PTEN+ cases.

To investigate whether expression of EGFR and its downstream targets were associated with clinical outcome, we performed univariate Cox regression analysis. Expression of PTEN (HR: 2.4; 95% CI: 1.2–5.0) and pAKT (HR: 2.2; 95% CI: 1.0–4.6) were significantly associated with a worse LRC (Table 4). Locoregional control was also significantly worse in case of lymph node metastases (HR: 5.7; 95% CI: 2.0–16.3) and in case of extranodal spread (HR: 5.0; 95% CI: 2.4–10.6). The multivariate Cox regression analysis for LRC showed that PTEN (HR: 2.2; 95% CI: 1.1–4.6), extranodal spread (HR: 2.7; 95% CI: 1.2–6.5) and the presence of lymph node metastasis (HR: 5.7; 95% CI: 1.2–27.4) were all independent prognostic factors for LRC. Kaplan–Meier survival analysis showed that patients with PTEN-positive HNSCCs had worse LRC (Figure 1B). Because PTEN and N-status are independent predictors for increased risk for locoregional failure (Table 4), we performed a stratified analysis that revealed that also within the subset of N+ patients, PTEN positivity identified patients with significantly worse LRC (*P*<0.001) (Figure 1C). In the current study population, the primary anatomical site of the tumour had no significant effect on LRC (data not shown) and no significant interactions were found between the anatomical site of the tumour and IHC staining with respect to LRC, indicating that the effect of PTEN on LRC did not depend on tumour site.

### Increased PTEN expression induces increased radioresistance *in vitro*

**Figure 2:**
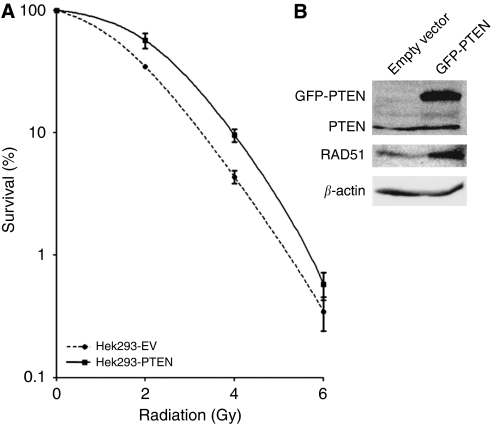
(**A**) Clonogenic assay, comparing the radiosensitivity of Hek293 cell line stabile transfected with an empty vector or with a plasmid overexpressing phosphatase and tensin homologue deleted on chromosome 10 (PTEN). (**B**) Western blot analysis for PTEN, RAD51 and *β*-actin. Notice that overexpression of PTEN is associated with overexpression of Rad51 in the stabile transfected PTEN cell line.

Our data based on IHC suggests that increased PTEN expression might affect the radiosensitivity of tumour cells. To investigate whether PTEN expression directly affected radiosensivity, we overexpressed the PTEN protein in Hek293 cells (Figure 2B) and tested the sensitivity to radiation *in vitro* by *in vitro* clonogenic assays. The PTEN-overexpressing Hek293 cells clearly showed increased radiation resistance compared with the empty vector control (Figure 2A).

## Discussion

The role of EGFR in oncogenesis is widely accepted, as activation of the receptor is known to result in important cancer hallmarks such as proliferation and resistance to apoptosis (Hanahan and Weinberg, 2000). Numerous studies reported on the prognostic value of overexpression of EGFR in many tumour types including HNSCC. The different studies on EGFR in HNSCC are unable to reach consensus on the effect of EGFR overexpression on the clinical outcome (Supplementary Table 2). Reasons for the inconsistency in the IHC could be the different antibodies used, no standard cutoff percentage for dichotomising the data and no consensus on the localisation of staining in the cell. In HNSCC, the percentage of EGFR-positive cases varies from 10 to 90% (Supplementary Table 2). Compared with these studies, the percentage of EGFR-positive cases in our study is low (12%). The association between EGFR positivity and low-stage tumours is unusual. We hypothesise that this could be related to the selection of our patient series, that is, only patients who received primary surgery and postoperative radiotherapy were included.

The total number of EGFR-positive cases with either complete or incomplete circumferential immunostaining of the tumour cell membranes was 19% (27 of 140 cases). However, FISH analysis to determine EGFR gene copy number changes revealed that only complete circumferential immunostaining of the tumour cell membranes using the EGFR113 antibody was a very specific and sensitive marker for amplification of the EGFR gene. As IHC stainings are more routinely performed than FISH analysis in many institutes, the antibody and the protocol that we used could very well be used as a pseudomarker for amplification. The frequency that we found is in good agreement with the previously described amplification frequency of EGFR in HNSCC (Freier *et al*, 2003; Koynova *et al*, 2005; Temam *et al*, 2007).

Recently, both hrHPV positivity and p16 positivity in HNSCC have been reported as very strong prognostic markers for clinical outcome (Chung and Gillison, 2009). In addition, EGFR expression has been inversely associated with the presence of HPV (Kong *et al*, 2009). To determine whether HPV positivity is related with LRC in our series and associated with EGFR expression, using a semi-quantitative HPV16-/HPV18-specific PCR, as well as the GP5+/6+ and CPI/IIg consensus PCR as reported previously (Wisman *et al*, 2006), only two cases were high-copy HPV16 positive (data not shown). Immunostaining for p16 revealed expression in six cases (3 of 25 oropharyngeal and 3 of 79 oral carcinomas). In the current literature, p16 positivity and hrHPV positivity is mainly reported in carcinomas in the oropharyngeal area (Machado *et al*, 2010). The relative low number of HPV-positive and p16-positive cases in our series is most probably because of the fact that our study contains only 25 oropharyngeal carcinomas (18%).

In addition, we observed a significant association between PTEN and pAKT. Phosphatase and tensin homologue deleted on chromosome 10 was originally identified as an antagonist of the AKT pathway, as expression resulted in decreased activation of AKT (Sarbassov *et al*, 2005). We found that PTEN expression is not only associated with higher pAKT levels but also with nuclear localisation of pERK, both indicators of EGFR pathway activation. These data suggested that the EGFR pathway is more activated in PTEN-positive than in PTEN-negative HNSCC. The positive correlation between PTEN and pAKT has recently been described in ovary cancer (Wang *et al*, 2005; de Graeff *et al*, 2008), breast cancer (Panigrahi *et al*, 2004) and malignant melanoma (Slipicevic *et al*, 2005). The AKT was reported to become activated by PDK1 (on threonine 308) and by PDK2 (on serine 473), in case AKT is bound to PIP3. Phosphatase and tensin homologue deleted on chromosome 10 dephosphorylates PIP3 to PIP2, by which AKT cannot bind to it anymore and will therefore not be activated by these kinases (Sarbassov *et al*, 2005). An alternative pathway for the phosphorylation of AKT is the integrin pathway. It has been reported that the integrin-linked kinase (ILK) is able to phosphorylate AKT at ser473, independent of the PI3K pathway (Persad *et al*, 2000). Tumours that are positive for PTEN seem to have an alternative way of activating AKT and ERK, and the ILK pathway might well be that way.

In our study, both positive PTEN and positive pAKT staining showed a significantly increased risk for LRR. In addition, the presence of extranodal spread and lymph node metastases revealed a significantly increased risk. A multivariate analysis for LRR for these four variables revealed that PTEN status, N-stage and extranodal spread are all independent significant factors, indicating that the PTEN status could have an additive value in determining the prognosis of advanced-stage HNSCC, next to the already used clinical factors of N-stage and extranodal spread.

To support the results of our IHC staining, we performed a clonogenic assay on Hek293 cells, transfected with an overexpression plasmid for PTEN, and compared it with the same cell line transfected with an empty vector. These *in vitro* data are in line with our findings in the patient series, that is, higher expression of PTEN leads to reduced radiosensitivity. To our knowledge, we are the first to publish this effect of PTEN on radiosensitivity *in vitro*.

The PTEN/MMAC1 was originally described as a tumour suppressor in a large variety of malignant tumours (Li *et al*, 1997; Panigrahi *et al*, 2004; Guney *et al*, 2007). In numerous cancer cell lines and primary tumours, mutations in the PTEN gene and loss of heterozygosity or homozygous deletions have been found (Cairns *et al*, 1997; Guldberg *et al*, 1997; Guney *et al*, 2007). As described earlier, PTEN regulates AKT activity through dephosphorylation of PIP3 (Sarbassov *et al*, 2005). More recently, two alternative functions for PTEN associated with its nuclear localisation were described (Shen *et al*, 2007). First, PTEN has a role in maintaining chromosomal stability by forming a complex with the CENP-C protein that binds and stabilises centromeres. In addition, PTEN was shown to bind to the promoter region of the double strand break (DSB) repair protein Rad51, resulting in upregulation of Rad51 (Shen *et al*, 2007; Yin and Shen, 2008). Interestingly, Rad51 expression was also upregulated in our radiation-resistant, PTEN-overexpressing transfectants (Figure 2B), which was in good agreement with the reported study (Shen *et al*, 2007). The protein Rad51 has been studied extensively and described to repair DSB through homologous recombination (Raderschall *et al*, 2002; Shen *et al*, 2007; Klein, 2008). The Rad51^−/−^ mice are not viable (Lim and Hasty, 1996) and DT40 Rad51 knockdown cells have been reported to accumulate chromosomal breaks during the replication and arrest in the G2/M phase of the cell cycle (Sonoda *et al*, 1998). Furthermore, overexpression of the Rad51 protein has been reported to increase the radioresistance of cell lines (Lundin *et al*, 2003). The association between PTEN overexpression and both worse LRC in patients as well as radioresistance in cell lines (this study) might therefore be explained through the overexpression of Rad51.

In conclusion, PTEN-positive HNSCC have a worse LRC when treated with primary surgery and postoperative radiotherapy. In these tumours, the AKT pathway is more frequently activated than in PTEN-negative tumours. A PTEN-overexpressing HEK293 cell line is more radiation resistant than the empty vector-transfected control cells, and in this overexpressing cell line RAD51 is upregulated. We therefore propose that the mechanism by which PTEN-positive HNSCC have a worse LRC is through upregulation of RAD51, leading to better DSB repair.

## Figures and Tables

**Table 1:** Patient characteristics

**Characteristic**	***N* (%)**
Total	140 (100)
	
*Age*	
Median (range), years	60 (24–90)
	
*Gender*	
Female	46 (33)
Male	94 (67)
	
*Primary location*	
Larynx	28 (20)
Hypopharynx	8 (6)
Oropharynx	25 (18)
Oral cavity	79 (56)
	
*T status*	
T1	6 (4)
T2	30 (22)
T3	34 (24)
T4	70 (50)
	
*N status*	
N0	50 (36)
N+	90 (64)
N1	32 (23)
N2a	1 (1)
N2b	42 (30)
N2c	13 (9)
N3	2 (1)
	
*Stage*	
I	1 (1)
II	21 (15)
III	17 (12)
IV	101 (72)
	
*Resection margins*	
Free	43 (31)
Not free	97 (69)
	
*Extra nodal spread*	
Yes	51 (36)
No	89 (64)

**Table 2:** Cross-tables showing the correlations between the expression levels of different proteins

**(a)**	**pEGFR**		
	**Low**	**High**	**Total**
*EGFR*			
Low	78 (63)	45 (37)	123
High	6 (35)	11 (65)	17
Total	84	56	140
			
Pearson's *χ*^2^-value is 4.9; *P*=0.027			
			
**(b)**	**pEGFR**		
	**Low**	**High**	**Total**
*pERK*			
Negative	78 (66)	40 (34)	118
Positive	6 (27)	16 (72)	22
Total	84	56	140
			
Pearson's *χ*^2^-value is 11.6; *P*=0.001			
			
**(c)**	**PI3K p110**		
	**Low**	**High**	**Total**
*pERK*			
Negative	63 (53)	55 (47)	118
Positive	19 (86)	3 (14)	22
Total	82	58	140
			
Pearson's *χ*^2^-value is 8.3; *P*=0.004			
			
**(d)**	**PI3K p110**		
	**Low**	**High**	**Total**
*Her2*			
Negative	79 (61)	50 (39)	129
Positive	3 (27)	8 (73)	11
Total	82	58	140
			
Pearson's *χ*^2^-value is 4.8; *P*=0.028			
			
**(e)**	**PTEN**		
	**Low**	**High**	**Total**
*pERK*			
Negative	86 (71)	32 (29)	118
Positive	11 (50)	11 (50)	22
Total	97	43	140
			
Pearson's *χ*^2^-value is 4.6; *P*=0.033			
			
**(f)**	**PTEN**		
	**Low**	**High**	**Total**
*pAKT*			
Low	81 (77)	24 (23)	105
High	16 (46)	19 (54)	35
Total	97	43	140
			
Pearson's *χ*^2^-value is 12.2; *P*<0.001			
			

Abbreviations: EGFR=epidermal growth factor receptor; HER2=human epidermal growth factor receptor 2; pAKT=phosphorylated protein kinase B; pEGFR=phospho-EGFR; pERK=phosphorylated extracellular signal-regulated kinase; PI3K=phosphoinositide 3-kinase; PTEN=phosphatase and tensin homologue deleted on chromosome 10.

**Table 3:** Cross-table EGFR FISH and EGFR IHC

	**EGFR FISH**		
	**Normal**	**Amplification**	**Total**
*EGFR IHC*			
Low	101 (99)	1 (1)	102
High	3 (19)	13 (81)	16
			
Total	104	14	118

Abbreviations: EGFR=epidermal growth factor receptor; FISH=fluorescent *in situ* hybridisation; IHC=immunohistochemistry.

Fischer exact test: *P*<0.001.

Specificity, 97% sensitivity, 93%.

**Table 4:** Cox regression analysis on IHC and clinical factors, both univariate and multivariate

**Cox regression**	***N* (%)**	**Hazard ratio (HR) for LRR (95% CI; *P*-value)**
*Univariate*		
EGFR		
Low	123 (89)	1
High	17 (11)	0.5 (0.1–2.0; *P*=0.32)
		
pEGFR		
Low	84 (60)	1
High	56 (40)	1.2 (0.6–2.6; *P*=0.58)
		
PI3K		
Low	87 (62)	1
High	53 (38)	0.6 (0.3–1.4; *P*=0.25)
		
PTEN		
Low	97 (69)	1
High	43 (31)	2.4 (1.2–5.0; *P*=0.019)^*^
		
pAKT		
Low	105 (75)	1
High	35 (25)	2.2 (1.0–4.6; *P*=0.043)^*^
		
pERK		
Negative	118 (84)	1
Positive	22 (16)	1.2 (0.5–3.0; *P*=0.65)
		
HER2		
Low	129 (92)	1
High	11 (8)	1 (0.2–4.2; *P*=0.99)
		
Lymph nodes		
Negative	50 (36)	1
Positive	90 (64)	5.7 (2.0–16.3; *P*=0.001)^*^
		
ENS		
No	89 (64)	1
Yes	51 (36)	5 (2.4–10.6; *P*<0.001)^*^
		
Surgical margins		
Free	43 (31)	1
Not free	97 (69)	1.4 (0.6–3.2; *P*=0.37)
		
*Multivariate*		
PTEN		
Low	97 (69)	1
High	43 (31)	2.2 (1.1–4.6; *P*=0.03)^*^
		
Lymph nodes		
Negative (N0)	50 (36)	1
Positive (N+)	90 (64)	5.7 (1.2–27.4; *P*=0.03)^*^
		
ENS		
No	89 (64)	1
Yes	51 (36)	2.7 (1.2–6.5; *P*=0.02)^*^

Abbreviations: CI=confidence interval; EGFR=epidermal growth factor receptor; ENS=extra nodal spread; HER2=human epidermal growth factor receptor 2; IHC=immunohistochemistry; pAKT=phosphorylated protein kinase B; pEGFR=phospho-EGFR; pERK=phosphorylated extracellular signal-regulated kinase; PI3K=phosphoinositide 3-kinase; PTEN=phosphatase and tensin homologue deleted on chromosome 10.

^*^P<0.05.
