# Supplementary material for: The phosphatase and tensin homologue deleted on chromosome 10 mediates radiosensitivity in head and neck cancer
Source: Br J Cancer. 2010 May 25;102(12):1778–85. doi: 10.1038/sj.bjc.6605707 (PMC2883706; doi:10.1038/sj.bjc.6605707)
Supplement: Supplementary Figure 1 [file 6605707x1.doc]

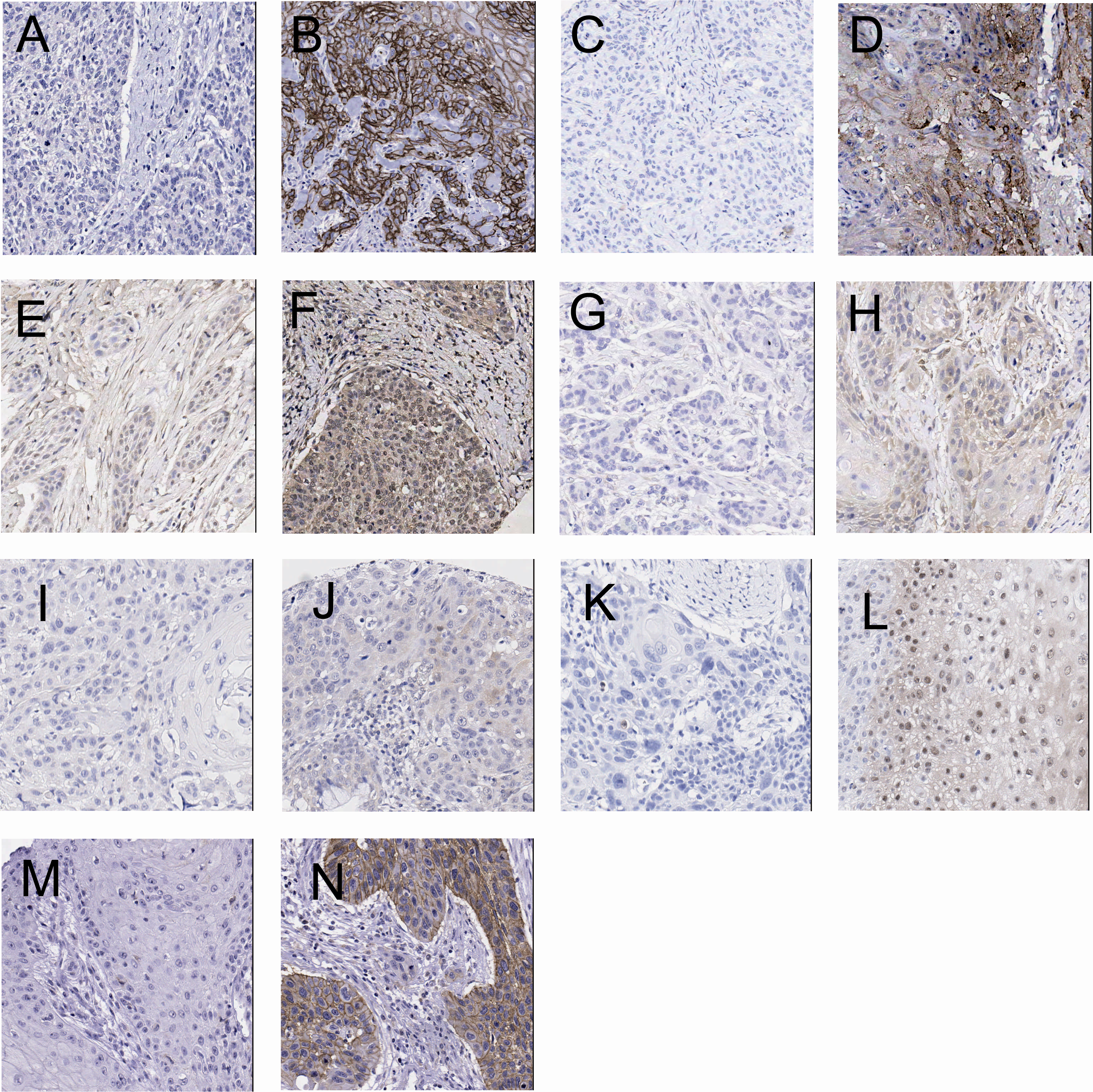


Figure 1 Examples of negative and positive cases in the immunohistochemical staining for EGFR (A,B), phoshoEGFR (C,D), PI3K p110 (E,F), PTEN (G,H), phoshoAKT (I,J), phosphoERK (K,L) and HER2 (M,N)
