# Supplementary material for: The phosphatase and tensin homologue deleted on chromosome 10 mediates radiosensitivity in head and neck cancer
Source: Br J Cancer. 2010 May 25;102(12):1778–85. doi: 10.1038/sj.bjc.6605707 (PMC2883706; doi:10.1038/sj.bjc.6605707)
Supplement: Supplementary Table 1 [file 6605707x2.pdf]

Supplementary Table 1. An overview of the Pearson correlation coefficients of the correlations between the different IHC stainings, and clinical factors

	EGFR	pEGFR	PI3K p110	PTEN	pAKT	pERK	Her2	N-Stage	T-Stage	Stage	ENS
EGFR	1,000	,187*	-,046	,037	,038	,080	-,109	-,088	-,182*	-,257**	-,145
pEGFR	,187*	1,000	-,154	,120	,034	,288**	-,076	,061	-,187*	-,176*	-,042
PI3K	-,046	-,154	1,000	-,151	,151	-,244**	,186*	-,099	,163	,005	-,004
PTEN	,037	,120	-,151	1,000	,295**	,181*	-,137	,012	,037	-,001	,075
pAKT	,038	,034	,151	,295**	1,000	,068	,015	,120	,113	,028	,214*
pERK	,080	,288**	-,244**	,181*	,068	1,000	,020	-,047	-,195*	-,126	-,082
Her2	-,109	-,076	,186*	-,137	,015	,020	1,000	,107	-,010	,122	,110
N-Stage	-,088	,061	-,099	,012	,120	-,047	,107	1,000	-,029	,302**	,564**
T-Stage	-,182*	-,187*	,163	,037	,113	-,195*	-,010	-,029	1,000	,436**	,038
Stage	-,257**	-,176*	,005	-,001	,028	-,126	,122	,302**	,436**	1,000	,338**
ENS	-,145	-,042	-,004	,075	,214*	-,082	,110	,564**	,038	,338**	1,000

* Significant at the 0.05 level. ** Significant at the 0.01 level.
