# Supplementary material for: The phosphatase and tensin homologue deleted on chromosome 10 mediates radiosensitivity in head and neck cancer
Source: Br J Cancer. 2010 May 25;102(12):1778–85. doi: 10.1038/sj.bjc.6605707 (PMC2883706; doi:10.1038/sj.bjc.6605707)
Supplement: Supplementary Table 2 [file 6605707x3.pdf]

Supplementary Table 2. Overview of publication on the prognostic value of EGFR expression in HNSCC

author	Year	antibody	Source	Scored	n=	site	treatment	cut off	LC	OS	Ref Nr.
Ang	2002	31G7	Zymed Lab.	MOD	155	HN	RT	MED	p=0.0031	p=0.0006	[1]
Bentzen	2005	EGFR.113	Novocastra	M	304	HN	RT	>0%	NS	NS	[2]
Carracedo	2008	2-18C9	Dako	M	47	P/L	S	MED	NS	NS	[3]
Chang	2008	NM	Zymed Lab.	M+C	151	P	RT	INT	p=0.047	NM	[4]
Demiral	2004	NM	Dako	MOD	31	L	RT	5%	p=0.03	NM	[5]
Eriksen	2004	2-18c9	Dako	M	336	HN	RT	50%	NM	NM	[6]
Eriksen	2005	2-18c9	Dako	M	209	L	RT	50%	NS	NM	[7]
Fischer	2008	EGFR.113	Novocastra	M+C	299	HN	S/RT/CRT	60%	NS	NS	[8]
Gupta	2002	H11	Dako	M	38	OP	CRT	INT	NS	NS	[9]
Hofman	2008	31G7	Invitrogen	M+C	176	HN	S	50%	p=0.011	p=0.023	[10]
Huang	2009	EGFR.113	Novocastra	M+C	172	O	S	50%	NM	NM	[11]
Laimer	2007	NM	Dako	M	109	O/OP	S/C/RT	INT	NM	p=0.05	[12]
Preuss	2008	31G7	Zymed Lab.	M	73	OP	S/PORT	1.	NS	NS	[13]
Ryott	2009	31G7	Zymed Lab.	M	78	O	S/R + S	INT	NS	NS	[14]
Sheikh Ali	2008	EGFR.113	Novocastra	M	65	HN	C+S		NS	NS	[15]
Smid	2006	EGFR.113	Novocastra	M	165	O	PORT	INT	NS	NS	[16]

M = membrane, C = cytoplasm, MOD = mean optical density, HN = all HNSCC, L = Larynx, P = Pharynx, OP = Oropharynx, O = Oral cavity, RT =radiotherapy, S = surgery, CRT = chemo-radiotherapy, PORT = postoperative radiotherapy, MED = median, INT = intensity, 1. = 4 groups based on intensity and %, NS = not significant, NM = not mentioned.

Reference List

[1] Ang KK, Berkey BA, Tu X, et al. Impact of epidermal growth factor receptor expression on survival and pattern of relapse in patients with advanced head and neck carcinoma. Cancer Res 2002;62:7350-6.

[2] Bentzen SM, Atasoy BM, Daley FM, et al. Epidermal growth factor receptor expression in pretreatment biopsies from head and neck squamous cell carcinoma as a predictive factor for a benefit from accelerated radiation therapy in a randomized controlled trial. J Clin Oncol 2005;23:5560-7.

[3] Carracedo DG, Astudillo A, Rodrigo JP, Suarez C, Gonzalez MV. Skp2, p27kip1 and EGFR assessment in head and neck squamous cell carcinoma: prognostic implications. Oncol Rep 2008;20:589-95.

[4] Chang AR, Wu HG, Park CI, Jun YK, Kim CW. Expression of epidermal growth factor receptor and cyclin D1 in pretreatment biopsies as a predictive factor of radiotherapy efficacy in early glottic cancer. Head Neck 2008;30:852-7.

[5] Demiral AN, Sarioglu S, Birlik B, Sen M, Kinay M. Prognostic significance of EGF receptor expression in early glottic cancer. Auris Nasus Larynx 2004;31:417-24.

[6] Eriksen JG, Steiniche T, Askaa J, Alsner J, Overgaard J. The prognostic value of epidermal growth factor receptor is related to tumor differentiation and the overall treatment time of radiotherapy in squamous cell carcinomas of the head and neck. Int J Radiat Oncol Biol Phys 2004;58:561-6.

[7] Eriksen JG, Steiniche T, Overgaard J. The role of epidermal growth factor receptor and E-cadherin for the outcome of reduction in the overall treatment time of radiotherapy of supraglottic larynx squamous cell carcinoma. Acta Oncol 2005;44:50-8.

[8] Fischer C, Zlobec I, Stockli E, et al. Is immunohistochemical epidermal growth factor receptor expression overestimated as a prognostic factor in head-neck squamous cell carcinoma? A retrospective analysis based on a tissue microarray of 365 carcinomas. Hum Pathol 2008;39:1527-34.

[9] Gupta AK, McKenna WG, Weber CN, et al. Local recurrence in head and neck cancer: relationship to radiation resistance and signal transduction. Clin Cancer Res 2002;8:885-92.

[10] Hofman P, Butori C, Havet K, et al. Prognostic significance of cortactin levels in head and neck squamous cell carcinoma: comparison with epidermal growth factor receptor status. Br J Cancer 2008;98:956-64.

[11] Huang SF, Chuang WY, Chen IH, Liao CT, Wang HM, Hsieh LL. EGFR protein overexpression and mutation in areca quid-associated oral cavity squamous cell carcinoma in Taiwan. Head Neck 2009.

[12] Laimer K, Spizzo G, Gastl G, et al. High EGFR expression predicts poor prognosis in patients with squamous cell carcinoma of the oral cavity and oropharynx: a TMA-based immunohistochemical analysis. Oral Oncol 2007;43:193-8.

[13] Preuss SF, Weinell A, Molitor M, et al. Survivin and epidermal growth factor receptor expression in surgically treated oropharyngeal squamous cell carcinoma. Head Neck 2008;30:1318-24.

[14] Ryott M, Wangsa D, Heselmeyer-Haddad K, et al. EGFR protein overexpression and gene copy number increases in oral tongue squamous cell carcinoma. Eur J Cancer 2009.

[15] Sheikh Ali MA, Gunduz M, Nagatsuka H, et al. Expression and mutation analysis of epidermal growth factor receptor in head and neck squamous cell carcinoma. Cancer Sci 2008;99:1589-94.

[16] Smid EJ, Stoter TR, Bloemena E, et al. The importance of immunohistochemical expression of EGFr in squamous cell carcinoma of the oral cavity treated with surgery and postoperative radiotherapy. Int J Radiat Oncol Biol Phys 2006;65:1323-9.
